# Cardiovascular Effects of Pulmonary Exposure to Single-Wall Carbon Nanotubes

**DOI:** 10.1289/ehp.9688

**Published:** 2006-12-04

**Authors:** Zheng Li, Tracy Hulderman, Rebecca Salmen, Rebecca Chapman, Stephen S. Leonard, Shih-Houng Young, Anna Shvedova, Michael I. Luster, Petia P. Simeonova

**Affiliations:** 1 Toxicology and Molecular Biology Branch and; 2 Pathology and Physiology Research Branch, Health Effects Laboratory Division, National Institute for Occupational Safety and Health, Morgantown, West Virginia, USA

**Keywords:** atherosclerosis, inflammatory cytokines, mitochondrial DNA damage, nanomaterials, nanotoxicology, oxidative stress

## Abstract

**Background:**

Engineered nanosized materials, such as single-wall carbon nanotubes (SWCNT), are emerging as technologically important in different industries.

**Objective:**

The unique physical characteristics and the pulmonary toxicity of SWCNTs raised concerns that respiratory exposure to these materials may be associated with cardiovascular adverse effects.

**Methods:**

In these studies we evaluated aortic mitochondrial alterations by oxidative stress assays, including quantitative polymerase chain reaction of mitochondrial (mt) DNA and plaque formation by morphometric analysis in mice exposed to SWCNTs.

**Results:**

A single intrapharyngeal instillation of SWCNTs induced activation of heme oxygenase-1 (HO-1), a marker of oxidative insults, in lung, aorta, and heart tissue in HO-1 reporter transgenic mice. Furthermore, we found that C57BL/6 mice, exposed to SWCNT (10 and 40 μg/mouse), developed aortic mtDNA damage at 7, 28, and 60 days after exposure. mtDNA damage was accompanied by changes in aortic mitochondrial glutathione and protein carbonyl levels. Because these modifications have been related to cardiovascular diseases, we evaluated whether repeated exposure to SWCNTs (20 μg/mouse once every other week for 8 weeks) stimulates the progression of atherosclerosis in ApoE^−/−^ transgenic mice. Although SWCNT exposure did not modify the lipid profiles of these mice, it resulted in accelerated plaque formation in ApoE^−/−^ mice fed an atherogenic diet. Plaque areas in the aortas, measured by the *en face* method, and in the brachiocephalic arteries, measured histopathologically, were significantly increased in the SWCNT-treated mice. This response was accompanied by increased mtDNA damage but not inflammation.

**Conclusions:**

Taken together, the findings are of sufficient significance to warrant further studies to evaluate the systemic effects of SWCNT under workplace or environmental exposure paradigms.

The most attractive properties of nanomaterials for medical and technologic applications, including their small size, large surface area, and high reactivity, are also the main factors for their potential toxicity (reviewed by Donaldson et al. 2001; Oberdörster et al. 2005). Based on inhalation studies with ambient ultrafine particles, it has been predicted that nanosized particles will have higher pulmonary deposition and biological activity compared with larger particles (reviewed by Oberdörster et al. 2005). Thus, some nanosized materials may induce not only damage at the deposition site but also distant responses as a result of their translocation and/or reactivity throughout the body (Oberdörster et al. 2005). In this respect, epidemiologic and experimental studies have suggested an association between respiratory exposure to ambient ultrafine particles (including particles with a diameter < 100 nm) and the progression of cardiovascular disease (reviewed by Brook et al. 2004).

Engineered carbon nanomaterials, including carbon nanotubes, have elicited a great deal of interest recently because of their unique electronic and mechanical properties. Single-wall carbon nanotubes (SWCNTs) have a diameter ranging from 0.7 to 1.5 nm, with lengths > 1 μm. Initial toxicologic studies demonstrated that intratracheal or pharyngeal instillation of SWCNT suspension in mice caused a persistent accumulation of carbon nanotube aggregates in the lung, followed by the rapid formation of pulmonary granulomatous and fibrotic tissues at the site (Lam et al. 2004; Shvedova et al. 2005). The unique physical characteristics and the pulmonary toxicity of SWCNTs raised concerns that respiratory exposure to these materials may be associated with systemic toxicities. In the present studies, we demonstrate that lung instillation of SWCNT is associated with a dose-dependent increase in oxidative vascular damage manifested by heme oxygenase (HO)-1 gene activation and mitochondrial alterations. Because these types of oxidative modifications are considered to play a role in atherogenesis, we further evaluated the effects of SWCNT respiratory exposure on atherosclerosis progression in ApoE^−/−^ transgenic mice, a widely used model of human atherosclerosis.

## Materials and Methods

### Animals

Male FVB/N-TgN (*Ho1-luc*) Xen mice were obtained from Xenogen Corp. (Alameda, CA). These mice carry a fusion of a 15-kb mouse HO-1 promoter with modified firefly luciferase cDNA (Promega pGL-3) and were used to assess HO-1 activation. Male C57BL/6J and B6.129P2-Apoe^tm1Unc^ (ApoE^−/−^) mice were obtained from Jackson Laboratory (Bar Harbor, ME). The ApoE^−/−^mice lack apolipoprotein E (a high-affinity ligand for lipoprotein receptors), consequently have elevated plasma levels of cholesterol and triglycerides, and develop atherosclerotic plaques in a fashion similar to humans (Reddick et al. 1994). Mice were between 2 and 3 months of age when exposures began, and were fed an irradiated commercial mouse chow diet (LM-485; Harlan, Indianapolis, IN). For some of the atherosclerosis studies, ApoE^−/−^ mice were fed a high fat diet (15.8% fat; TD 88051; Harlan), which exacerbates the atheroma formation. All mice were provided tap water *ad libitum* and were housed in ventilated cages on autoclaved hardwood chip bedding. Sentinel mice were free of endogenous pathogens. Animal room conditions included HEPA-filtered air, controlled temperature and humidity, and a 12-hr light/dark cycle. Animal care and use procedures, including sacrifice by carbon dioxide asphyxiation, were conducted in accordance with the *Public Health Service Policy on Humane Care and Use of Laboratory Animals* (Office of Laboratory Animal Welfare 2002) and the *Guide for the Care and Use of Laboratory Animals* (Institute of Laboratory Animal Resources 1996); these procedures were approved by the National Institute for Occupational Safety and Health institutional animal care and use committee. All mice used in these studies were treated humanely and with regard for alleviation for suffering.

### General experimental design

To screen for systemic oxidative effects of SWCNT exposure, *Ho1-luc* reporter mice or C57BL/6 mice (at least 4 mice per treatment) were exposed to SWCNT in doses of 10–40 μg/mouse by single intrapharyngeal instillation and were sacrificed at time points including 1, 7, 28, and 56 days after exposure. These experimental settings were selected to correspond to previous pulmonary toxicity studies (Shvedova et al. 2005). Pulmonary toxicity has been characterized with early but acute and transient inflammatory response, granuloma formation around SWCNT agglomerates, and a fibrogenic response. To evaluate SWCNT effects on atherosclerosis progression, a chronic process, ApoE^−/−^ mice (10/treatment) were exposed by pharyngeal aspiration to a medium dose of SWCNT (20 μg/mouse) via multiple exposures (once every other week for 8 weeks). In all studies the control animals were exposed by pharyngeal aspiration to vehicle [sterile phosphate-buffered saline (PBS)].

### Particles and particulate instillation

SWCNT (CNI, Houston, TX) were produced by the high pressure carbon monoxide proportionation process (HiPco) technique (Scott et al. 2003; Scott and Smalley 2003) and purified by acid treatment to remove metal contaminants (Gorelik et al. 2000). Their physicochemical characteristics, including chemical content, mean diameter, and surface area, were reported previously (Shvedova et al. 2005). Ultrafine carbon black (UfCB) particles (Printex 90, Degussa, Germany), used as a control material, were a gift from G. Oberdörster (University of Rochester, Rochester, NY). Suspensions of SWCNTs and UfCBs were prepared by sonification using a Branson Sonifier 450 (Branson Ultrasonics Corp., Danbury, CT) in PBS for 3 min (at 400 W) at room temperature before animal exposure. For particulate administration, we used the pharyngeal aspiration technique (Rao et al. 2003). Briefly, mice were anesthetized with isoflurane (Abbott Laboratories, North Chicago, IL) and placed on a slant board, and the tongue was gently held in full extension while a 60-μL suspension of particles was pipetted onto the base of the tongue. This technique provides for a wide dissemination of particles in a peribronchiolar pattern within the alveolar region (Rao et al. 2003).

### Tissue and blood collection

Food was removed from the cage for ~ 8 hr before sacrifice. Whole blood samples were drawn from the inferior vena cava into EDTA-containing tubes. The plasma was collected and stored for later analysis. The heart and vascular tree were perfused with ice-cold PBS containing heparin (10 U/mL) for the atherosclerotic lesion analysis. Animals used for evaluation of oxidative stress were perfused with ice-cold PBS containing 20 mM butylated hydroxytoluene (BHT) to prevent further oxidation. For DNA damage studies, the aortas were retrieved and rapidly frozen in liquid nitrogen and stored at −80°C before analysis. For histopathologic studies, the aorta and heart were harvested and stored in buffered formalin (10%), or the brachiocephalic artery (BCA) was dissected and snap-frozen in Tissue-Tek OCT-compound (Sakura Finetek, Torrance, CA) to be used for cryostat sections.

### Measurement of luciferase activity

Pieces (10 mg each) of lung, heart, and aorta from *Ho1-luc* mice were removed, placed in 0.2 mL tissue lysis buffer (Promega, Madison, WI), chopped into fine pieces, and left overnight at 4°C on a shaker. After brief centrifugation, supernatants of the tissue lysates were collected and luciferase activity was measured using a commercial kit (Promega) and a MicroLumat Plus LB 96V luminometor (EG&G Berthold, Bad-Wildbad, Germany).

### Quantitative polymerase chain reaction (QPCR) for evaluating mitochondrial (mt) DNA damage

The QPCR assay, which evaluates the average lesion frequency per strand for the two template strands in the segment of interest, was conducted according to a protocol described previously (Ballinger et al. 1999, 2000) with slight modifications. Briefly, genomic DNA was isolated using the Qiagen genomic tip 20G kit (Qiagen Inc., Valencia, CA). The DNA concentration was measured using the PicoGreen dsDNA quantitation kit (Molecular Probes, Eugene, OR). QPCR of extra long amplification product was carried out using the Expand 20 kb PCR^plus^ System (Roche, Mannheim, Germany). A mouse 12-kb mtDNA fragment was amplified using the following primers: F5′-AGAGAT-TTCTCTACACCTTCGAATTTGC-3′ and R 5 ′ - A T T T A A T T T G A G G G T G A -CGGGCGG-3′. The PCR reaction system contained 100 ng DNA as a template. Amplification consisted of an initial cycle of denaturation (92°C, 2 min) followed by 25 cycles of denaturation (92°C, 30 sec), annealing (65°C, 30 sec), and extension (68°C, 15 min), with a final extension (68°C, 10 min) cycle. A short (180 bp) fragment from the D-loop part of mtDNA was amplified using the following primers: F5′-TAATCAGCC-CATGACCAACA-3′ and R5′-GATTGGG-TTTTGCGGACTAA-3′. Ten microliters of PCR products were applied to gel electrophoresis (0.8% agarose gel, 75 V, 1 hr). Gels were stained by ethidium bromide, imaged using the Alphaimager System (Alpha Innotech Corp., San Leandro, CA), and quantified by scanning laser densitometry.

### Aortic mitochondria isolation

Mitochondria were isolated from fresh aortas by differential centrifugation (Young et al. 2005). Briefly, tissues were homogenized in isotonic buffer containing 10 μg/mL protease inhibitors (aprotinin, phenylmethylsulphonylfluoride, leupeptin, sodium orthovanadate) and BHT (5 mM) at 4°C. Tissue homogenates were centrifuged at 1,500 × *g* and 4°C for 15 min, and the supernatants were collected. The supernatants were centrifuged at 10,000 × *g* for 30 min at 4°C, and the mitochondria were collected in the pellet. Mitochondrial protein concentration was measured in BSA-free isolation buffer by a Lowry Assay (Bio-Rad Corp., Hercules, CA). Samples were stored at −70°C.

### Mitochondrial oxidative stress assays

Mitochondrial reduced glutathione/oxidized glutathione (GSH/GSSG) ratios were measured by a Bioxytech GSH/GSSG-412 colorimetric assay kit (OxisResearch, Portland, OR) with minor modifications. Briefly, 10 μL 1-methyl-2-vinylpyridinium trifluoromethane-sulfonate was added to scavenge for GSH during the mitochondrial isolation. The reaction mixture was added to a 96-well microtiter plate at a volume of 50 μL and incubated at room temperature for 5 min. NADPH was added to each well, and the change in absorbance at 412 nm was recorded in a microplate reader (Molecular Device, Sunnyvale, CA) for 3 min. GSH and GSSG levels were normalized to protein concentrations and the GSH/GSSG ratio was calculated. Mitochondrial carbonyl groups were detected by slot blot (Amersham Biosciences, Piscataway, NJ). Briefly, 10 μL mitochondrial protein was derivatized with 2,4-dinitrophenylhydrazine and blotted to polyvinylidene fluoride membrane (Amersham Biosciences, Piscataway, NJ). The dirivatized carbonyl groups, detected by rabbit anti-dinitrophenol (1:500; Molecular Probes) and horseradish peroxide–conjugated anti-rabbit IgG (1:1,000) were visualized by an ECL system (Amersham, Piscataway, NJ). Band densities were quantified by densitometry (ImageQuant, Molecular Dynamics).

### Quantification of atherosclerosis

We determined the extent of aortic atherosclerotic lesions by the *en face* method, as described previously (Simeonova et al. 2003). Briefly, the aorta was fixed in 10% neutral buffered formalin, opened longitudinally, and stained with Sudan IV. Aorta images were captured with a Sony DXC-960MD three-chip-coupled device color video camera (Sony, Tokyo, Japan), connected to an Olympus SZX12 dissection microscope (Olympus, Melville, NY). Among the individual segments of the same aorta, the smallest coefficient of variation occurred in the segment that contained the most extensive lesions (Palinski et al. 1994); thus the thoracic part of the aorta was used for quantitative assessment. Quantitative analysis of the lesion size was performed using a Simple PCI C-imaging system (Compix Inc., Cranberry Township, PA).

Plaque formation in brachiocephalic arteries was quantified as described by Teupser et al. (2003). Briefly, BCAs were sectioned from distal to proximal at a 10-μm thickness. Atherosclerotic lesions luminal to the internal elastic lamina were quantified in eight equidistant oil red O–stained sections 200, 300, 400, 500, 600, 700, and 800 μm from the branching point of the BCA into the carotid and subclavian arteries. The images were captured by a Sony DXC-960MD three-chip-coupled device color video camera (Sony) connected to an Olympus AX70 microscope (Olympus) and analyzed by Simple PCI C-imaging system (Compix Inc., Sewickley, PA). For immunostaining analysis, we applied specific rat anti-mouse monoclonal antibodies for Mac-3 (a marker of monocytes/macrophages) and vascular cell adhesion molecule 1 (VCAM-1) (both from BD PharMingen, San Diego, CA) in an immunostaining protocol conducted on acetone-fixed transverse cryosections; the positive staining was evaluated as described previously (Warren et al. 2005).

### Plasma cytokine levels

Analysis of cytokines from plasma samples was conducted using a mouse inflammation cytometric bead array kit and a FACSCalibur flow cytometer (both from BD Biosciences, San Diego, CA) following the manufacturer’s instructions. Standard curves were determined for each cytokine from the range of 20–5,000 pg/mL. The lower detection limit of the assay is 2.5–52.7 pg/mL, depending on types of cytokines. The following cytokines were measured: interleukin-6 (IL-6), monocyte chemoattractant protein-1 (MCP-1), interferon-γ (IFN-γ), tumor necrosis factor-α (TNF-α), and IL-12.

### Statistical analysis

All experiments were replicated, and representative findings are shown. Differences among treatment groups were detected by analysis of variance. Differences in which the *p*-value was < 0.05 were considered statistically significant. When significant differences were detected, post hoc comparisons between treatment groups were examined with the Holm-Sidak method.

## Results

### HO-1 activation in cardiovascular tissue

**Figure 1:**
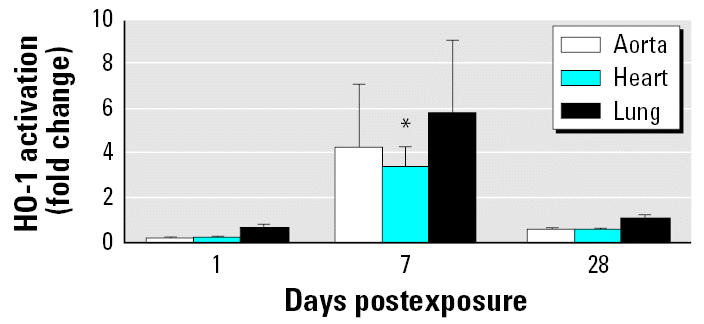
Time course of HO-1 activation in the lung and cardiovascular system of *Ho1-luc* reporter mice exposed to SWCNTs (40 μg) or vehicle (PBS). The results are presented as a fold change in the luciferase activities of the tissue from SWCNT-exposed mice compared with the respective tissue from PBS-exposed mice. Each value represents the mean ± SE of five mice. *p < 0.05.

We conducted initial screening for extrapulmonary effects using *Ho1-luc* reporter transgenic mice. To evaluate *HO-1* gene expression, a surrogate biomarker of oxidative stress (Prawan et al. 2005; Rao et al. 2003), we measured the activation of tissue luciferase in *Ho1-luc* reporter transgenic mice. HO-1 demonstrated a trend for activation in the lung, aorta, and heart tissue 7 days after SWCNT exposure; the activity declined approximately to control levels by 28 days after exposure (Figure 1).

### Aortic mitochondrial oxidative damage

**Figure 2:**
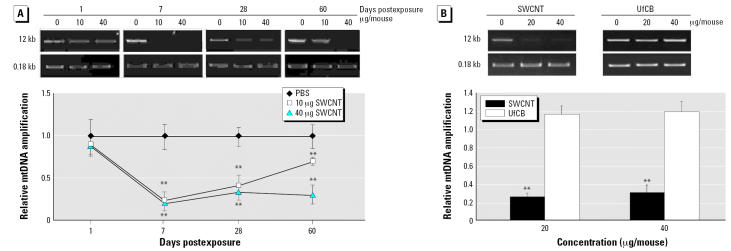
Aortic mtDNA damage in C57BL/6 mice exposed to SWCNTs shown as representative gel images of the long (12 kb) and short fragment (0.18 kb) mtDNA amplification products and quantitative analyses of the mtDNA damage (a reduction in the amplification) presented as a fold difference between each SWCNT-treated group and the vehicle-treated group. (*A*) Mice were treated with SWCNTs (0, 10, or 40 μg) and aortas were collected at 7, 28, and 60 days postexposure. (*B*) Mice were treated with SWCNTs (0, 20, or 40 μg) or UfCBs (0, 20, or 40 μg) and aortas were collected 60 days postexposure. The 12 kb mtDNA expression was normalized to 0.18 kb mtDNA expression. Each value represents the mean ± SE of four mice. **p < 0.01.

Furthermore, we conducted specific evaluation of vascular oxidative perturbations in C57BL/6 mice. Because circular mtDNA is highly susceptible to oxidative damage and mtDNA damage has been suggested as an initiating event in atherogenesis (Ballinger et al. 2000, 2002), we assessed whether pulmonary SWCNT exposure induces aortic mtDNA damage. Significant reduction in mtDNA amplification, reflecting an increase in mtDNA damage, was observed in the aortic tissue of C57BL/6 mice exposed to SWCNT (10 and 40 μg/mouse) at days 7, 28, and 60 after a single exposure, compared with age-matched, vehicle-treated control mice (*p* < 0.01) (Figure 2A). Maximum mtDNA damage occurred 7 days after exposure in both treatment groups and persisted in the high-dose treatment group 60 days after exposure. Amplification of a small fragment of the D-loop of the mitochondrial genome (Figure 2A), as well as nuclear 18S rRNA (data not shown), was not affected by the exposure. Accordingly, the amplification of the large mtDNA fragment was normalized to the amplification of the small fragment. In contrast to SWCNT, UfCB pharyngeal instillation did not result in mtDNA damage in the aortic tissue at comparable doses of exposure (Figure 2B).

**Figure 3:**
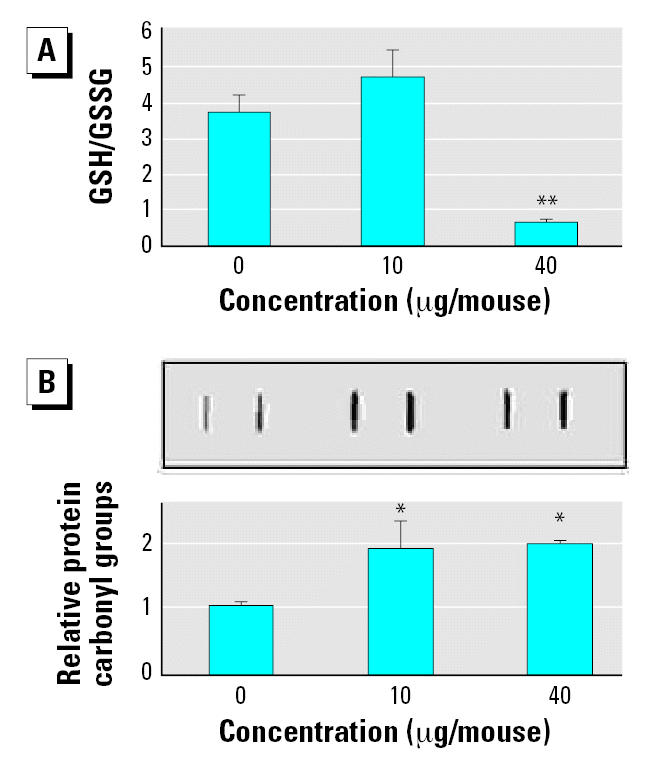
Oxidative modifications in aortic mitochondria freshly isolated from C57BL/6 mice treated with SWCNTs (0, 10, or 40 μg) 7 days post-exposure. (*A*) GSH/GSSG ratio of aortic mitochondria. (*B*) Protein carbonyl groups in the aortic mitochondrial proteins (10 μg) shown by representative dot blot images and densitometric analysis. The results are presented as a fold change in the aortic protein carbonyl levels of SWCNT-exposed mice compared with PBS-exposed mice. Each value represents the mean ± SE of four mice. *p < 0.05. **p < 0.01.

Changes in mitochondrial GSH levels are also manifestations of oxidative stress (Suliman et al. 2004). The aortic mitochondrial GSH/GSSG ratios were significantly reduced 7 days after pulmonary exposure to a single high dose of SWCNT (Figure 3A). Oxidative stress also may lead to oxidation of amino acid residues on proteins, forming protein carbonyls (Dalle-Donne et al. 2003). Consistently, significant accumulation of aortic mitochondrial protein carbonyl groups occurred in SWCNT-exposed mice compared with the age-matched vehicle-exposed mice at both exposure levels (*p* < 0.05) (Figure 3B).

### Atherosclerosis evaluation in ApoE^−/−^ mice

**Figure 4:**
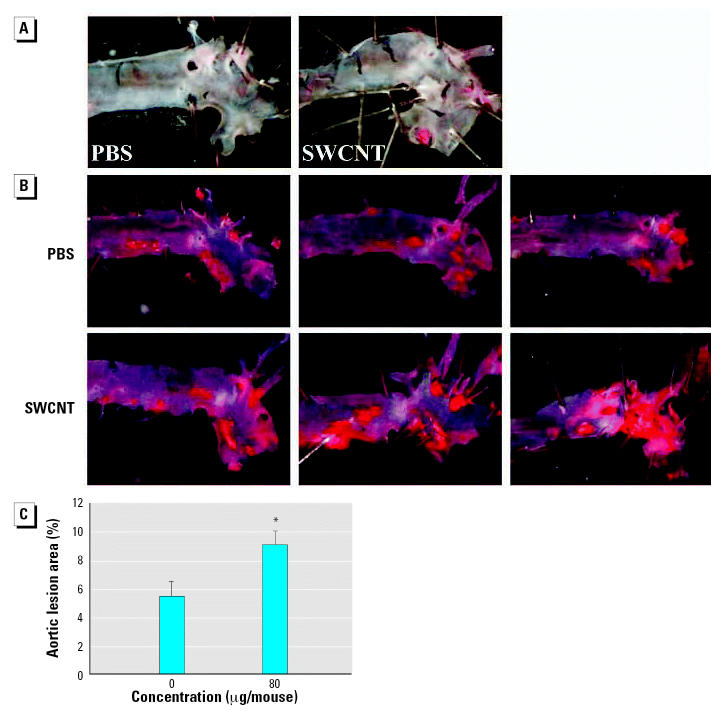
Atheroma formation in the aortas of ApoE^−/−^ mice repeatedly exposed to SWCNTs (20 μg/mouse every other week for 8 weeks). Photomicrographs of representative Sudan IV-stained thoracic aortas of mice on regimen 1 (*A*) and regimen 2 (*B*). (*C*) Morphometric analysis of the atherosclerotic lesions in the thoracic aortas of mice fed on regimen 2. Each value represents the mean ± SE of 10 mice. *p < 0.05.

**Figure 5:**
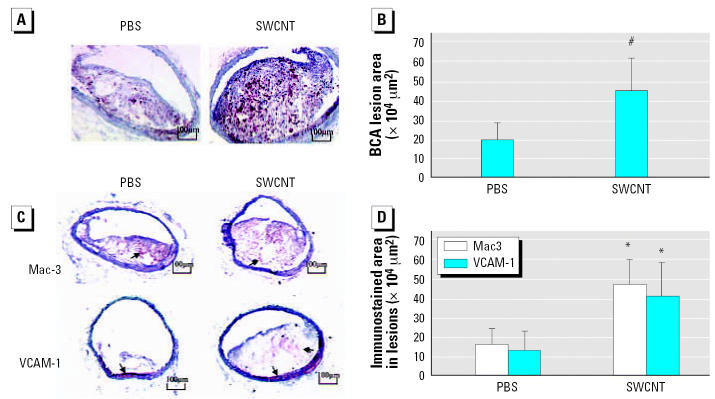
Atheroma formation in the BCA of ApoE^−/−^ mice repeatedly exposed to SWCNTs (20 μg/mouse every other week for 8 weeks) and fed regimen 2. (*A*) Representative images of oil red O–stained serial sections of BCA. (*B*) Quantification analysis of the atherosclerotic lesions in seven sections of BCA (200, 300, 400, 500, 600, 700, and 800 μm proximal from its branching site into the carotid and subclavian arteries). (*C*) Images of representative BCA sections immunostained for Mac-3 and VCAM-1; the arrow indicates positive immunostaining. (*D*) Quantification analysis of the immunostained sections. For (*B*) and (*D*), each value represents the mean ± SE of 10 mice. *p < 0.05. ^#^*p* < 0.001.

The effects of pulmonary SWCNT exposure on atherosclerosis development were evaluated in ApoE^−/−^ mice. ApoE^−/−^ mice were exposed chronically to PBS or SWCNT (four instillations of SWCNT at 20 μg/mouse every other week for 8 weeks) and fed a regular chow diet (regimen 1) or a fat chow diet for 4 weeks, followed by regular chow for 4 weeks (regimen 2). Vehicle-exposed ApoE^−/−^ mice on regimen 1 had lower plasma cholesterol (*p* ≤ 0.01) and minimal plaque occurrence in the aorta compared with the mice on regimen 2. The body weights, as well as plasma cholesterol, glucose, and lactate dehydrogenase (LDH) levels were comparable in SWCNT-and vehicle-exposed mice under both diet regimens (Table 1). Plaque formation, although appearing slightly increased after exposure to SWCNT, compared with the vehicle exposure in mice on regimen 1, was not quantified because the size of the plaques was not large enough for meaningful analysis or comparisons to be made (representative images presented in Figure 4A). However, in mice on regimen 2, the aortic morphometric *en face* analysis demonstrated a significant increase in the plaque areas in SWCNT-exposed mice compared with the vehicle-treated mice (*p* < 0.05) (Figure 4B, C). Histologic assessment of the lesion size in the BCAs of these mice confirmed that SWCNT exposure accelerated atherosclerosis progression (Figure 5). The BCA was quantified at seven sections: 200, 300, 400, 500, 600, 700, and 800 μm proximal from its branching site into the carotid and subclavian arteries. SWCNT exposure was associated with considerably larger atherosclerotic lesion area at each BCA location examined (representative images shown in Figure 5A). The overall lesion area of the BCA was significantly higher in the SWCNT-exposed mice than in the PBS-treated mice (mean ± SE, 447,239 ± 164,242 μm^2^ and 192,911 ± 90,755 μm^2^, respectively; *p* < 0.001; Figure 5B).

To assess the influence of SWCNT exposure on inflammatory characteristics of the plaque, serial sections of the BCA were immunostained with antibodies to Mac-3 (Ralph et al. 1983) and VCAM-1 (Matheny et al. 2000). Consistent with the larger size of the plaques, the number of macrophages and VCAM-1–positive cells were proportionally higher in the BCA plaques from SWCNT-exposed mice compared with plaques in PBS-treated mice (Figure 5C, D). In the evaluated sections, we found no evidence that SWCNT exposure resulted in increased immunostaining in the vascular wall, which was not affected by plaque formation.

**Figure 6:**
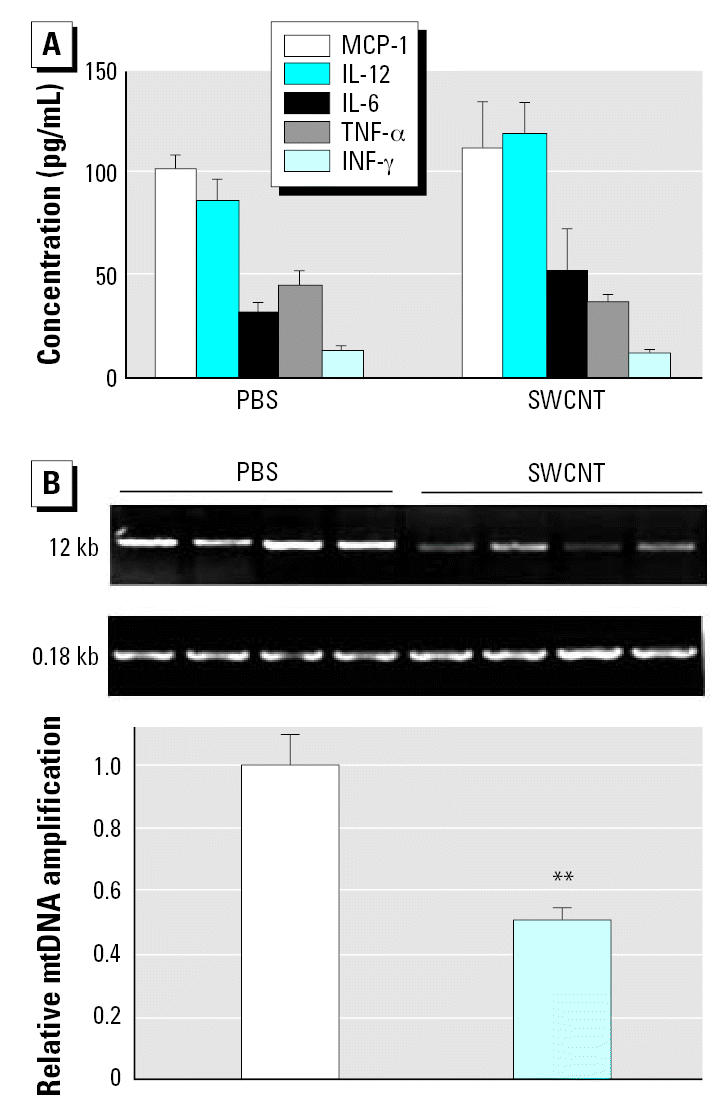
Evaluation of systemic inflammation and aortic mitochondrial DNA damage in ApoE^−/−^ mice repeatedly exposed to SWCNTs (20 μg/mouse every other week for 8 weeks) and fed regimen 2. (*A*) Plasma levels of MCP-1, IL-12, IL-6, TNF-α, and INF-γ (pg/mL); each value represents the mean ± SE of 10 mice. (*B*) Representative gel images of the long (12 kb) and short (0.18 kb) fragment mtDNA amplification products and quantitative analyses of the mtDNA damage presented as a fold difference between the SWCNT-treated group and the vehicle-treated group. The 12-kb mtDNA expression was normalized to 0.18-kb mtDNA expression; each value represents the mean ± SE of 4 mice. **p < 0.01.

We also evaluated the involvement of systemic inflammation and aortic mtDNA damage in SWCNT-related atherosclerosis progression. The plasma MCP-1, IL-12, IL-6, TNF-α, and IFN-γ levels were not significantly different in ApoE^−/−^ mice treated with PBS or SWCNT (Figure 6A). However, the aortas of SWCNT-exposed ApoE^−/−^ mice demonstrated significantly increased mtDNA damage.

## Discussion

These studies demonstrate that SWCNTs, under the described conditions, have the potential to influence cardiovascular diseases. The effects were manifested as distressed mitochondrial homeostasis represented by mtDNA damage, GSH depletion, and increased protein carbonyl formation in aortic tissue. Mitochondrial components have been reported to be highly susceptible to oxidative stress, mediated by metabolic defects and environmental insults (Madamanchi et al. 2005), and mitochondrial dysfunction is emerging as an important pathophysiologic factor in a number of cardiovascular diseases including atherosclerosis (Binkova et al. 2001). Oxidative alterations of mitochondria result in compromised metabolic processes, such as oxidative phosphorylation, which can trigger endothelial dysfunction, a leading mechanism in atherosclerosis progression (Choksi et al. 2004). Altered endothelial activities lead to a series of events including vasoconstriction, increased adhesiveness resulting in inflammatory cell infiltration, and platelet-thrombus formation (Cai and Harrison 2000; Libby 2000a, 2000b). Combination of multiple cardiovascular risk factors that work through similar processes, such as mitochondrial dysfunction, may lead to synergistic acceleration of atherosclerosis progression and precipitation of its complications. Consistently, atherosclerosis was accelerated in SWCNT-exposed ApoE^−/−^ mice primed with a high-fat diet. Although SWCNT-exposed ApoE^−/−^ mice did not have altered lipid profiles, they had exacerbated plaque development in the aorta and BCAs.

In addition to the well-established cardiovascular risk factors, such as high cholesterol levels, diabetes mellitus, and hypertension, many nontraditional risk factors, including concomitant infections, systemic autoimmune diseases, and arsenic exposure, have been suggested to influence atherosclerotic process and precipitate disease complications (Libby 2000b; Ross 1999; Simeonova and Luster 2004). In this respect, epidemiologic and experimental studies have recently found a positive association between air pollution (i.e., ultrafine particulates) and adverse cardiovascular outcomes, particularly in high risk individuals such as those with preexisting chronic pulmonary or cardiovascular diseases (Brook et al. 2004; Künzli et al. 2005; Peters et al. 2004; Pope et al. 2004). Consistently, hyperlipidemic rabbits exposed to PM_10_ (particulate matter ≤ 10 μm in aerodynamic diameter) or ApoE^−/−^ mice exposed to PM_2.5_ (≤ 2.5 μm ) develop advanced coronary and/or aortic atherosclerosis (Chen and Nadziejko 2005; Sun et al. 2005). Evidence from human and toxicologic exposure studies suggest that oxidative stress and inflammation is most likely involved in particle-mediated cardiovascular effects (Barclay et al. 2005; Brook et al. 2004; Sun et al. 2005). Although the role of mitochondrial distress in atherosclerosis related to respiratory particle exposure has not been well explored, cigarette smoke and hypercholesterolemia have been shown to result in mtDNA damage and greater plaque formation (Knight-Lozano et al. 2002).

Pulmonary exposure to SWCNTs may induce cardiovascular effects either directly or indirectly through mitochondrial oxidative perturbations, which can result in altered vessel homeostasis. SWCNT-induced lung pathophysiologic responses are associated with deposition of particle agglomerates and histopathologic alterations in the lung. Hypothetically, it is possible that individual SWCNTs can translocate from the lung into the systemic circulation causing direct cardiovascular endothelial dysfunction. It has been reported that nanoparticles treated with albumin and/or surfactant proteins cross the alveolo-capillary barrier to gain access to the systemic circulation (Kato et al. 2003; Oberdörster et al. 2005). The proximity between epithelial type I and endothelial cell caveolar membrane structures might play a role in the particle translocation mechanisms (Heckel et al. 2004). Because SWCNTs are not well recognized and cleared by lung macrophages (Kagan et al. 2006; Shvedova et al. 2005), nanotubes, dispersed or disintegrated from the agglomerates, may persist in the alveolar space, which will facilitate their access into the systemic circulation. In contrast to SWCNTs, a similar dose of UfCB agglomerated particles, which are readily phagocytized by alveolar macrophages, did not produce lung fibrosis or granulomas (Shvedova et al. 2005) or induce cardiovascular mtDNA damage. It is also possible that indirect processes are responsible for the cardiovascular effects induced by SWCNT exposure. This could occur if either mediators, released from the lung into the systemic circulation, or hypoxemia, associated with altered pulmonary function seen after SWCNT exposure (Shvedova et al. 2005), lead to oxidative modifications and low-level systemic inflammation. In the present study, several inflammatory mediators known to play a role in atherosclerosis were measured in the ApoE^−/−^ mice evaluated for the atherosclerotic lesions. Although SWCNT exposure was associated with atherosclerosis acceleration, we did not observe significant differences in the plasma levels of IL-6, IL-10, MCP-1, TNF-α, or IFN-γ. Furthermore, SWCNT exposure was not related to increased inflammation in the vascular wall before formation of the plaques. A third hypothesis for SWCNT exposure–mediated cardiovascular effects is through platelet activation in the lung circulation. The pulmonary circulation is considered a site for platelet formation (Martin et al. 1983; O’Sullivan and Michelson 2006), and it has been demonstrated that SWCNT can directly activate platelet aggregation *in vitro* (Radomski et al. 2005). Furthermore, transforming growth factor-β1(TGF–β1), which is involved in platelet activation (Hoying et al. 1999), has been found to be significantly increased in the lung of SWCNT-treated mice (Shvedova et al. 2005), and the time course of its increase paralleled the occurrence of cardiovascular mitochondrial dysfunction. Although a link between activated platelets and cardiovascular mitochondrial distress has not been clearly established, it is well understood that that both platelet activation and mitochondrial damage lead to endothelial dysfunction and atherosclerosis (Ballinger 2005; Ross 1993).

Overall, these initial studies demonstrate that respiratory exposure to high concentrations of mostly agglomerated SWCNTs provokes not only pulmonary toxicity but vascular effects related to mitochondrial oxidative modifications and accelerated atheroma formation. Taken together, the findings are of sufficient significance to warrant further studies to evaluate the systemic effects of SWCNTs under inhalation exposure paradigms more likely to occur in the workplace or environment, such as low-level chronic inhalation exposure. Studies in progress involving labeled SWCNTs, as well as detailed analysis of the role of lung platelet activation, will provide more insight into the mechanisms of the cardiovascular mitochondrial dysfunction in SWCNT-treated animals.

## Figures and Tables

**Table 1:** Body weight (BW), plasma cholesterol, triglyceride, glucose, and LDH in ApoE^−/−^ mice exposed to PBS or SWCNT (four instillations of SWCNT at 20 mg/mouse every other week for 8 weeks) and fed regimen 1 or regimen 2 diets.

Regimen	Treatment	Start BW	End BW	Total cholesterol	Triglyceride	Glucose	LDH
1	PBS	25.9 ± 0.17	25.2 ± 0.76	606 ± 68	131 ± 27	340 ± 24	142 ± 39
	SWCNT	24.3 ± 0.20	25.0 ± 0.56	628 ± 69	131 ± 25	351 ± 36	114 ± 4
2	PBS	19.8 ± 0.57	23.7 ± 0.84	1,541 ± 336**	122 ± 15	271 ± 17	413 ± 40**
	SWCNT	20.8 ± 0.79	23.7 ± 0.72	1,674 ± 157**	70 ± 8	266 ± 10	318 ± 23**

Each value represents the mean ± SE of four mice per group.

** *p* < 0.01 compared with regimen 1 and the same respiratory treatment.
